# The Effectiveness of Acceptance and Commitment Therapy on Quality of Life in a Patient with Myocardial Infarction: A Randomized Control Trial

**Published:** 2020-01

**Authors:** Leila Ahmadi Ghahnaviyeh, Bagherian Bagherian, Awat Feizi, Atefe Afshari, Firoozeh Mostafavi Darani

**Affiliations:** 1 Department of Health Education and Health Promotion, Isfahan University of Medical Sciences, Isfahan, Iran.; 2 Department of Psychiatry, School of Medicine, Isfahan University of Medical Sciences, Behavioral Sciences Research Center, Isfahan, Iran.; 3 Department of Statistics, School of Health, Isfahan University of Medical Sciences, Isfahan, Iran.; 4 Department of Health Education and Health Promotion, School of Health, Isfahan University of Medical Sciences, Isfahan, Iran.

**Keywords:** *Acceptance and Commitment Therapy*, *Myocardial Infarction*, *Quality of Life*, *Randomized Control Trial*

## Abstract

**Objective:** Acceptance and commitment therapy (ACT) interventions increase psychological flexibility and improve mental health and quality of life in patients with myocardial infarction.

Study design: A controlled clinical trial study was conducted to evaluate the efficacy of an ACT intervention in improving the quality of life in patients with MI in Isfahan, Iran.

**Method** **:** The present controlled clinical trial with a pre and post-test design was conducted on a statistical population consisting of patients with MI admitted to hospitals in Isfahan (n = 60) who were selected through sequential sampling based on the study inclusion criteria and were randomly divided into an intervention and a control group (n1 = n2 = 30). The case group received 8 weekly 90-minute sessions of ACT and the control group received no interventions. The pretest-posttest design was administered in both groups using a demographic questionnaire and the Minnesota Living with Heart Failure Questionnaire (MLHFQ) designed to assess the health status of patients with heart failure in terms of quality of life. The data obtained were analyzed in SPSS-20 using descriptive statistics and the ANCOVA.

**Results: **In this study, 2 general areas of quality of life, including physical and mental health, were examined in the patients. There was a significant increase in the quality of life and subscales of mental and physical health in the experimental group (p < 0.001).

**Conclusion: **Considering the effectiveness of ACT in improving quality of life in these patients, this method of intervention can be used as a complementary therapy in health care centers to reduce the side-effects experienced by these patients.

Myocardial infarction (MI) is one of the most prevalent diseases in many different societies. The incidence of MI has increased significantly in recent years. In the US, almost 650 000 new cases of acute MI and 450 000 cases of MI present to medical centers every year(1). MI is the most common cause of death in people aged over 35 in Iran( 2, 3). Damage to the heart caused by any disease affects the patient’s mental health in addition to causing physical disabilities and symptoms, and MI is no exception. MI is the main complication of coronary diseases and is given special attention due to its high mortality rate, complications, psychological problems, and negative effects on the patients’ quality of life(4).

Quality of life is a major factor that needs to be addressed by health and social professionals. The World Health Organization defines quality of life as satisfaction with various important aspects of life, including values, goals, standards, and individual interests, encompassing psychological, social, economic, and family(5). Some studies have shown that cardiovascular diseases affect patients’ quality of life as an independent factor(6).

Therefore, in addition to reducing their mortality and increasing their survival, efforts should also be made to give these patients an acceptable quality of life and to identify the factors affecting this variable. Due to the multiple stressors experienced by these patients in daily life, health-care planners should pay particular attention to the risk of reduced quality of life in patients with cardiovascular diseases(7).

Acceptance and commitment therapy (ACT) is a model that contributes significantly to the promotion of healthy behaviors and the improvement of mental health in patients with chronic diseases, especially in the form of group therapy. This model of therapy has attracted the greatest attention in research and clinical practice in cognitive-behavioral (group) therapy and in affecting behavior change and has thus been confirmed as efficient in most cases. ACT was first introduced by Steven Hayes at the University of Nevada, Reno, in 1987 with the acronym ACT. This method of intervention assumes that humans find many of their feelings, emotions, or inner thoughts disturbing and constantly seek to change these inner experiences or get free of them. These efforts to control are ineffective and further intensify the feelings, emotions, and thoughts the person has initially sought to avoid. The main goal of ACT is to increase psychological flexibility; that is, it seeks to develop an ability in the individual to make an actual decision between different choices rather than taking action or being forced into action merely to avoid the disturbing thoughts, feelings, memories, or desires(8).

In ACT, psychological flexibility develops through 6 main processes: (1) acceptance, (2) diffusion, (3) self as context, (4) commitment, (5) committed action, and (6) contact with the present moment, which are classified into 2 groups: (1) mindfulness and acceptance processes and (2) commitment and behavior change processes(9,10,11). These subjective experiences can include irrational and obsessive thoughts, anger, stress, fears, and social anxiety(12). A major advantage of this method of therapy over other psychotherapy approaches is that it considers motivational aspects along with cognitive ones to achieve a longer-lasting treatment effect(12). ACT has also been reported to increase the quality of life in patients with MI and to reduce their depression, stress, and anxiety(13,14,15). A higher acceptance of disease is associated with greater involvement in personal affairs, maintenance of compromise, reduction of confusion and disability, and improvement of psychological well-being(16). Therefore, acceptance of disease seems to be an appropriate strategy for helping patients better cope with their disease and improve their quality of life. 

Recent studies have highlighted the importance of the concept of disease acceptance and improvement in psychological functioning, quality of life, and adaptation to health problems that have characterized MI patients, as well as MI, being a worldwide and highly regarded disease affecting ACT interventions. The quality of life of patients with this disease has not been evaluated in Iran. Thus, the present study aimed to evaluate the effect of ACT intervention on quality of life of patients with MI in patients referred to Isfahan hospitals in 2015.

## Materials and Methods

***Trial Design***

The present clinical trial was conducted on a case and a control group with pre-test, post-test and 6-month follow-up. The patients were randomly divided into case (receiving ACT intervention in addition to the routine care) and control (receiving the routine care only) groups based on the hospital appointment system. The research setting consisted of Isfahan hospitals and the study population comprised of patients with MI admitted to these hospitals in 2015. The trial design was registered at the Iranian Registry of Clinical Trials under IRCT2016060828339N1 and was approved by the ethics committee of Isfahan University of Medical Sciences. 

***Participants***

A total of 60 patients were selected through simple sequential sampling based on the study inclusion and exclusion criteria and provided their MI diagnosis, which was confirmed by a specialist. The inclusion criteria for this study consisted of a history of at least one heart attack, age over 30, reading and writing literacy, and full consent to participate in the study. Individuals with other physical disorders such as acute renal disease or cancer who did not receive psychological treatment and also those with addiction or psychological illnesses such as psychosis who did not qualify for training sessions were excluded from the study (Figure2).

***Interventions***

The researcher introduced himself to the participants, conducted individual interviews with them, and briefed them on the study objectives and on how to complete the questionnaires. Moreover, the participants were ensured that their information would remain confidential.

 Also, the participants were ensured that they could withdraw from the study at any time. All the questionnaires were filled out individually, and to resolve any potential ambiguities, the researcher was present when the participants were completing the questionnaires. A pretest session was held and both groups responded to the questionnaires. To avoid bias in the results, the quality of life questionnaire was administered by a clinical psychologist (MSc) who helped with the research. The case group then received an ACT intervention in 8 weekly 90-minute sessions. The therapeutic approach intervention used in this study was the expanded version of the ACT intervention model, which has been approved in theory and practice by the Scientific Advisory Board(9,10). To verify the content validity of the intervention package and its compliance with the study objectives, the text of the package was given to 5 clinical psychologists fluent in English and familiar with the subject to confirm the accuracy of its translation. The text was then presented to several students in an informal class to confirm their accurate understanding of the content. 

Table 1 presents a summary of the content of the ACT intervention sessions. The patients were followed-up immediately after the intervention and 6 months later. To implement a similar method of intervention for the control group, 3 training sessions were held for the controls in the form of conferences in addition to their completion of the questionnaires and the prepared educational materials were presented to all the members of the control group. 

***Additional Trial Tools***

In addition to a demographic questionnaire, the following questionnaires were used in this study to collect the data.

The Minnesota Living with Heart Failure Questionnaire (MLHFQ) was developed to assess the health status of patients with heart failure in terms of the quality of life(17). The MLHFQ is a self-administered questionnaire containing 21 items. Each item is rated on a 6-point scale, ranging from 0 (indicating the best state) to 5 (indicating the worst state). The questionnaire has 2 parts: one part covers the physical activity limitations and the other part examines the psychological dimension of the patient's quality of life. These parts evaluate the effect of persistent physical symptoms such as shortness of breath, fatigue, peripheral oedema, sleep disorders, and the effect of psychological symptoms such as anxiety and depression on the patient's quality of life. The patient's score varies from 0 to 105, with higher scores reflecting a poorer health status. The results obtained from this questionnaire are often reported in 3 dimensions of health: the physical dimension, the psychological dimension, and the overall dimension. A Cronbach's alpha value of 92% has been reported for the questionnaire. Pearson's correlation coefficient of 78% has also been reported for the questionnaire through the pretest-posttest results(18).

***Statistical Analysis***

The data obtained in this study were analyzed in SPSS-20 using descriptive statistics, mean and standard deviation, ANOVA, paired t -test, independent t-test, Mann-Whitney’s test, and Chi-square test.

## Results

The demographic data showed that the mean age of the patients was 57.33 ± 9.42 in the case group and 55.1 ± 9.61 in the control group. The most frequent levels of education in both groups were incomplete secondary education (not possessing high school diploma) and a high school diploma. In terms of economic status, the results showed that in the case group, 20% of the participants had poor, 66.7% moderate, and 13.3% high economic status. In the control group, 26.7% had poor, 62.1% moderate and 11.2% high economic status, suggesting that the 2 groups were matched in terms of gender and the other variables studied. Table 2 demonstrates the descriptive indicators of the 2 groups.

**Figure 1:**
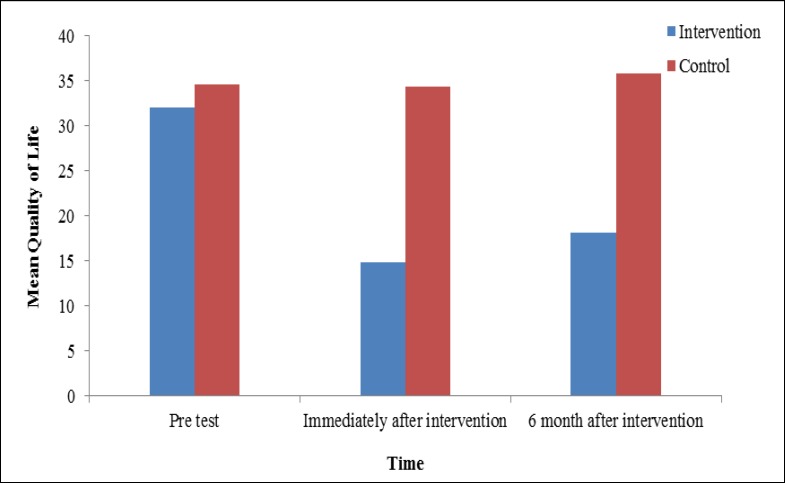
The Mean of Quality of Life After 6 Month

The mean score of overall quality of life in the intervention group immediately after the intervention and 6 weeks after the intervention decreased significantly, indicating an overall increase in the quality of life in the intervention group. However, the mean score of quality of life questionnaire was increased in the control group, indicating a decrease in their quality of life (Table 3 and Figure 1).

As shown in Table 3, the results of repeated measures ANOVA revealed a significant difference in the mean score of overall quality of life between the 2 groups during the follow-up (p < 0.001). The overall quality of life also showed a significant difference in each of the groups overtime (p < 0.001) (Figure1). The interaction of time and group was also statistically significant (p < 0.001). Changes in the response variable were different between the 2 groups at different points in time. Table 3 presents the descriptive indicators about the pretest, posttest, and 6-month follow-up scores of quality of life in the 2 groups and the ANOVA results.

In this study, 2 general areas of quality of life, including physical and mental health, were examined in the patients. As shown in Table 4, the results of repeated measures ANOVA revealed a significant difference between the 2 groups in terms of the mean scores of both the physical and mental dimensions of quality of life during the follow-up period (p < 0.001). A significant difference was also observed in the overall quality of life over time between the 2 groups (p < 0.001). The interaction of time and group was also statistically significant (p < 0.001). Changes in the response variable were different between the 2 groups at different points in time. 

Table 4 presents the descriptive indicators about the pretest, posttest, and 6-month follow-up scores of the physical and mental quality of life in the 2 groups and the ANOVA results. 

## Discussion

This study was conducted to determine the effectiveness of ACT intervention in increasing quality of life in patients with MI. The results showed that group therapy based on ACT intervention improved quality of life in patients with MI immediately after the intervention and 6 months later (p < 0.05).

This method of therapy also improves 2 general areas of quality of life: mental health and physical health. The physical quality of life in patients with MI is an important issue that affects patients after a heart attack. Having to sit or lie down to rest, difficulty in leaving the house, walking and climbing the stairs, sleep disorders due to physical problems, and shortness of breath are among the problems with which MI patients are faced. It appears that the physical and mental aspects of quality of life are affected by each other. To explain these findings, it can be argued that quality of life is a multidimensional construct. According to the WHO, quality of life includes not only physical and social aspects but also psychological aspects. Previous studies have found that coping strategies and psychological factors such as anxiety, depression, pain, and physical discomfort and their interference with daily activities contribute to quality of life in patients with chronic pain(5). Since no studies were found in databases on the effect of ACT on quality of life in patients with MI, at least in Iran, this section of the article, discusses the results of studies conducted on the effect of this method of intervention on other diseases, especially chronic diseases.

According to a study by Bazzazian et al, patients with higher experiential avoidance experience less positive emotions and life satisfaction and feel that their life is meaningless(13). Nevertheless, ACT aims to reduce experiential avoidance and increase psychological flexibility through accepting unavoidable, distressing and unpleasant feelings such as anxiety, cultivating mindfulness to neutralize excessive involvement with cognitions and identifying personal values related to behavioral objectives. Through ACT, while moving toward their valuable goals, the patients are also encouraged to establish full contact with their experiences without resistance and to accept them as they emerge without judgment of their truth or falsity. This practice increases the motivation for change despite the inevitable barriers and encourages the individual to make efforts for achieving his valuable life goals; this practice thus leads to an improved quality of life, especially in its psychological aspect (19). Mason et al. and Wicksell et al. also showed that, in addition to improving quality of life in patients with chronic diseases, ACT also reduces the pain experienced(13, 14). 

The reason for the success of ACT is that this approach focuses on functional processes underlying many impaired behavioral manifestations rather than focusing on the form or frequency of the symptoms that are characteristic of a disorder. An ACT intervention does not target a specific diagnostic category, rather it targets behavioral patterns that hinder a successful life. Instead of focusing on reducing the symptoms, the therapist seeks to improve the patient's overall quality of life with his cooperation. One of the reasons for the increased acceptance and understanding of disease in these patients may be that by clarifying their values and goals, they notice that instead of dealing with things they cannot change (eg, thoughts, feelings, memories, etc.), they should involve themselves in things they can change (eg, acting in harmony with their values, etc.).

The patients learn that they should move in harmony with their values and goals instead of dealing with unpleasant thoughts and the resulting anxiety and stress. Although this method of intervention directly deals with symptoms such as depression and distressing thoughts, it assumes that when the patient does not try to decrease his intrusive thoughts and feelings, and instead moves toward the goals he has set to match his/her life values, the symptoms are automatically improved. The cumulative results of this study and studies conducted by Twohig et al (2006) and Twohig (2007) showed that an ACT intervention is a very good therapeutic approach for dealing with psychological disorders, especially in patients who suffer from the psychological problems resulting from disease complications(19, 20). 

In the research of Marnie et al (2010) on the effectiveness of an acceptance and commitment therapy using self-help intervention for chronic pain, it was found that compared to controls, participants who completed the self-help book (case-group) showed improved quality of life and decreased anxiety. When data from all the treatment participants were pooled, those who completed the intervention showed statistically significant improvements (with large effect sizes) for acceptance, quality of life, satisfaction with life, and values illness. Medium effect sizes were found for improvements in pain ratings(21), which was consistent with the results of the present study, confirming the effect of acceptance and commitment therapy intervention training on chronic pain and chronic disease. Also, the study of Karlin and et al (2013) showed that ACT-D is an effective and acceptable treatment for older veterans treated in routine clinical settings, including those with high levels of depression(22), which confirmed the results of the present study.

Committed action is another process emphasized by ACT, and the proposed treatment protocol also highlighted its role. Encouraging the patients to clarify their values, to determine their goals, to predict the barriers, to ultimately commit to take actions which are in harmony with their values despite their illness, and to consider the limitations their sickness helps them achieve their goals and acquire happiness, while improving their understanding of the disease, increasing their control over their own life, and improving their quality of life.

## Limitation

This study did not examine other psychological aspects of patients such as anxiety, stress, and depression. Thus, it is recommended that they are further explored in future studies.

## Conclusion

The results obtained by this study showed that this therapeutic approach can significantly improve the quality of life in patients with MI, especially in its psychological aspect. Moreover, it was found that this psychological method could be useful in the hospitals in where services are provided to patients with myocardial infarction.

**Table 1:** A Summary of the Content of the ACT Intervention Sessions

**Session**	**Intervention/Content**
Introduction	Getting acquainted with the patients and establishing a good relationship with them to build trust for getting the questionnaires filled out properly, administering the demographic questionnaire and the pretest
First	Introducing the teaching expert, the group getting acquainted with each other and establishing a therapeutic relationship among themselves, introducing the Acceptance and Commitment Therapy intervention and its main objectives and pillars, setting ground rules for the entire sessions, providing information about heart failure and its complications, reviewing ways to control and prevent disease complications and their costs and benefits, providing psychological education, break and snacks, assigning the homework
Second	Reviewing experiences of the previous session and receiving feedback from the patients, discussing the experiences and assessing them, evaluating the patients’ tendency to change, understanding the patients’ expectations about the ACT intervention, fostering creative distress, break and snacks, summarizing the presented material and assigning the homework
Third	Reviewing experiences of the previous session and receiving feedback from the patients, identifying inefficient strategies and learning to control them and perceive their futility, explaining the concept of acceptance and its differences with concepts of failure, despair, denial and resistance, teaching that acceptance is a constant rather than logical process, discussing the problems and challenges of a heart attack, explaining how to avoid painful experiences and being mindful of the consequences of avoidance, discovering situations that have been avoided and contacting them through acceptance, defining coping and introducing effective and ineffective coping strategies, break and snacks, summarizing the presented material and an overview of the next session’s work, assigning the homework
Fourth	Reviewing experiences of the previous session and receiving feedback from the patients, break and snacks, behavioural commitment and obligation, introducing and explaining confused self-concept and its diffusion, the application of cognitive diffusion therapeutic approach, intervention in the performance of problematic chains of language and metaphors, discouraging the patients from wasting their time with thoughts and emotions, summarizing the presented material and an overview of the next session’s work, assigning the homework
Fifth	Reviewing experiences of the previous session and receiving feedback from the patients, showing the distinctions between the self, therapeutic experiences and behaviour, self as context, weakening the self-concept and self-expression. Through these practices, the participants learn to focus on their activities (such as breathing, walking, etc.) and be mindful of their state at all moments and learn to perceive their emotions, feelings and cognitions and to process them without judgment; that is, they learn to pay attention to their thoughts and emotions but not get attached to their content, break and snacks, summarizing the presented material and an overview of the next session's work, assigning the homework
Sixth	Reviewing experiences of the previous session and receiving feedback from the patients, identifying the patients’ values in life and focusing on these values, their elaboration and their power of choice, using mindfulness techniques with an emphasis on the present, break and snacks, summarizing the presented material and an overview of the next session’s work, assigning the homework
Seventh	Reviewing experiences of the previous session and receiving feedback from the patients, examining each patient’s values and giving further depth to the concepts previously taught, explaining the difference between values, goals and routine mistakes in the selection of values, discussing the potential internal and external barriers to the pursuit of values, the group members listing and sharing their most important values and the potential barriers to their pursuit, discussing the goals related to values and the characteristics of goals among the group, the group members identifying three of their most important values and determining the goals they wish to pursue in keeping with those values, determining the next steps for achieving those goals, break and snacks, summarizing the presented material and an overview of the next session’s work, assigning the homework.
Eighth	Understanding the nature of tendencies and commitment (teaching commitment to action), identifying behavioural models compatible with values and developing commitment to act on them, briefly discussing the concept of relapse and preparing to cope with it, reviewing the homework and summarizing the sessions with the patients, the group members sharing their experiences and discussing their gains and unmet expectations, the researcher expressing his gratitude to the patients for attending the sessions, administering the post-test.

**Table 2:** The Demographic Characteristics of the Patients Examined

**Factors**		**Case**	**Control**	**P -value** *
**Group**		**Frequency (Percentage)**		
Sex	Female	7 (23.3%)	6 (20%)	0.737
	Male	23 (76.7%)	24 (80%)	
Age (year)		57.33 ± 9.42	55.1 ± 9.61	0.654
History of hospitalization	No history of hospitalization	2 (6.7%)	3 (10%)	0.826
	Once	10 (33.3%)	9 (30%)	
	Twice or more	18 (60%)	18 (60%)	
Self -evaluation of the economic situation	Bad	6 (20%)	8 (26.7%)	0.007
	Moderate	20 (66.7%)	19 (62.1%)	
	Good	4 (13.3%)	3 (11.2%)	
Education status	Below high school education	16 (53.3%)	16 (53.3%)	0.277
	High school diploma	10 (33.3%)	9 (30%)	
	Associate’s degree	1 (3.3%)	39 (10%)	
	Bachelor’s degree and above	3 (10%)	2 (6.7%)	
Marital status	Single	2 (6.7%)	4 (13.3%)	0.181
	Married	28 (93.3%)	26 (86.7%)	

* The results showed the lack of a correlation between the demographic variables and the dependent variable (P ≥ 0.05).

**Table 3:** The Results of the Comparison between and between Intervention Group and Control Group in the Overall Quality of Life

**Factors**	**Time**	**Pre-test**	**Immediately ** **after ** **intervention**	**6 months after ** **theintervention**	**Time**	**Group**	**Time × ** **Group**
	**Group**	**Mean ± SD**			**P-Value**		
Quality of life	Intervention	32.03 ± 14.26	14.8 ± 7.23	18.16 ± 6.06	0.001	0.001	0.001
	Control	34.62 ± 16.34	34.31 ± 16.52	35.75 ± 16.01	0.09		
Group differences in any of the periods		0.519	0.001	0.001			

**Table 4:** The Results of the Comparison between Intervention Group and Case Group in the Physical and Mental Quality of Life

**Factors**	**Time**	**Pre-test**	**Immediately ** **after ** **intervention**		**6 months ** **after** **‏ the ** **intervention**	**Time**	**Grou** **p**	**Time ** **× ** **Group**
	**Group**	**Mean ± SD**				**P-Value**		
Physical quality of life	Intervention	18.7 ± 9.24		9.23 ± 5.39	11.53 ± 4.59	0.001	0.001	0.03
	Control	18.89 ± 8.94		18.58 ± 9.06	19.58 ± 8.87	0.34		
Group differences in any of the periods		0.934		0.001	0.001			
Psychological quality of life	Intervention	8.53 ± 4.98		3.53 ± 2.38	4.13 ± 2.43	0.001	0.001	0.001
	Control	9.6 ± 5.81		9.6 ± 5.81	9.83 ± 5.69	0.182		
Group differences in any of the periods		0.449		0.001	0.001			

**Figure 2:**
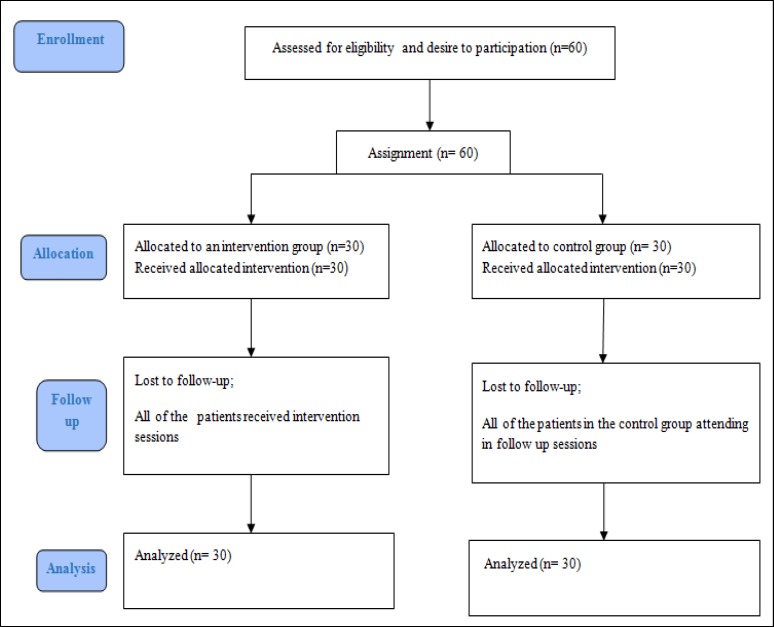
Consort Diagram of Contact Classification
